# Croupous Pneumonia in the Horse1Read before the Pennsylvania State Veterinary Medical Association, March 6, 1895.

**Published:** 1895-03

**Authors:** Arthur Salinger

**Affiliations:** Demonstrator of Surgery, Veterinary Department University of Pennsylvania


### CROUPOUS PNEUMONIA IN THE HORSE.[1]

BY ARTHUR SALINGER, V.M.D.,
DEMONSTRATOR OF SURGERY, VETERINARY DEPARTMENT UNIVERSITY OF PENNSYLVANIA.

THE consideration of croupous pneumonia is by no means new. A discussion of some of the more important symptoms and newer remedial agents, in the face of recent investigations, is, however, in place.

It is the object of this paper to call attention to the pathology, diagnosis, and treatment of acute lobar pneumonia. The synonyms for this disease are lobar, croupous or fibrinous pneumonia, pneumonitis and lung fever.

Pneumonia is one of the most widespread of all acute infectious diseases. There is scarcely an acute infectious disease so frequent in the horse as pneumonia. Climate does not seem to have much influence in the production of this disease; it pre-

[1] Read before the Pennsylvania State Veterinary Medical Association, March 6, 1895.

vails equally in cold and in hot countries. Cold has been thought to be one of the most important etiological factors, and it is undoubtedly true that the disease sometimes follows a sudden chilling or wetting; but in many cases it will be impossible to obtain any such history. Pneumonia frequently follows traumatisms of the chest, when it is known as contusion pneumonia.

A change of opinion has recently taken place since the development of bacteriology in regard to the etiology of croupous pneumonia. It is now unquestionably considered an infectious disease depending upon a specific micro-organism. What has furthermore given strength to this opinion among the medical fraternity is the fact that many epidemics of pneumonia have been described.

The diplococcus pneumonia of Fraenkel is the most constant organism found in croupous pneumonia, and it is now believed by the most competent authorities to be the specific agent of this disease. It is identical with the micrococcus which Pasteur and Sternberg found in the saliva of certain individuals and which produces septicæmia in the rabbit. The researches of Friedlaender, Weickselbaum and others show that it is by far the most constant organism in pneumonia. According to the dominant view pneumonia is an infectious disease caused by this diplococcus, which has its main seat and produces its chief effect on the lung, and can under favorable circumstances invade the pleura, meninges and endocardium. It is a widespread organism, at times present in the buccal secretions of healthy animals in the secretion of the nose and mouth. It may be demonstrated by treating the ordinary cover-glass preparation with glacial acetic acid and then washing off the acid, dropping on aniline oil and gentian violet, which is to be poured off and renewed two or three times. The organism is seen to be a somewhat elliptical, lance-shaped coccus occurring in pairs; hence the term diplococcus. It is usually encapsulated. Inoculation experiments upon dogs, guinea-pigs, and mice have proven successful, the germ producing the well-known pathological changes occurring in croupous pneumonia.

Morbid Anatomy. Since the time of Laennaec pathologists have divided the stages of pneumonia into three well-known divisions of engorgement, red hepatization and gray hepatization.

The stage of engorgement usually lasts but a short time—

from twelve to thirty-six hours. The lung tissue is deep red in color, more solid, and on section the surface is bathed in blood and serum. Crepitation is still present, although not so marked as in normal lung.

In the stage of red hepatization the lung tissue is firm, solid, and airless. On section the surface is dry, reddish-brown in color, and the deeply congested appearance of the first stage is absent. In this stage the lung is very friable and is readily broken by the finger. Careful inspection shows the surface to be distinctly granular and the air cells filled with fibrinous plugs. The smaller bronchi often contain fibrinous plugs. This stage usually lasts about two days.

Stage of gray hepatization is the first stage in the process of resolution. The exudate is softened, the cell elements are disintegrated and are rendered capable of absorption. The grayish appearance is due to the absorption of the hæmoglobin and red blood-corpuscles, with an increase in the number of white corpuscles. A point to which especial attention should be called is the almost frequent association of affection of the pleura. It can be stated that in every case of pneumonia where the inflammatory process reaches the periphery the pleura is affected.

The pleurisy may be either a dry one, pleuritis sicca, or a pleurisy with effusion may be present. We will have occasion again to refer to pleurisy with effusion in considering the symptomatology and prognosis of croupus pneumonia. It will be noticed that the right lung is more frequently affected than the left one.

Symptoms. This disease usually begins with a marked and prolonged chill, with rapidly rising temperature, frequently going from 103 to 106 degrees, with rapid pulse, marked depression, hebetude and anorexia. The extremities will be found to be cold. The pulse may be from 60 to 100 in a minute. In severe cases the conjunctiva may be seen to be jaundiced, due to profound blood alteration. Symptoms referable to the lungs manifest themselves at once. Respiratory movements are quickened and painful, due in the majority of cases to the early involvement of the pleura. The type of breathing is costal. The affected animal does not lie down, or if found in the recumbent position the animal will lie upon the affected side. The standing position of the horse is typical. The front legs are usually held far apart and the head is extended and carried low. The animal moves as quietly as possible, cautiously preventing unnecessary movements. Cough if present is slight, short and evidently painful. The most important diagnostic points are brought out by physical examination.

Percussion early in the first stage may give you a tympanitic note. This can only be observed if the case be seen early. This tympanitic note soon gives way to dulness on percussion, which shows the beginning of the stage of red hepatization or consolidation. In the third stage the percussion note tends toward clearing up, and in favorable cases returns to normal pulmonary resonance. If the pleura be affected, slight percussion over the affected area produces sharp pain.

On auscultation in the first stage we hear an increase of the respiratory murmur, accompanied by crepitant räles. This râle is heard only at the end of inspiration. As consolidation goes on the crepitant râle disappears, giving place to bronchial breathing. In the stage of resolution moist râles return, which are coarser than the râles first heard, and are known as the crepitus redux.

If pleurisy with effusion takes place at any stage of the disease, the respiratory symptoms will become aggravated. The dyspnœa already present will be changed to orthopnœa. Symptoms of cyanosis will begin to show themselves. It is obvious that if you have fluid at the base of a lung that is already affected by an inflammation of high grade the difficulty in breathing will become more marked. Hence the recognition of a pleurisy with effusion (no matter how slight the effusion) should be noticed at once

The urine has the characteristics of ordinary febrile urine—high specific gravity and usually alkaline in reaction. Albumin may be present, and the chlorides will be found to be markedly diminished.

The fever is typical, remaining high from the onset with very slight morning remissions. It remains so for five or six days, and in favorable cases declines, recovery usually taking place in from ten to fourteen days. The termination is usually abrupt, resembling crisis; the cough becomes looser, the respirations less rapid, the pulse slower and fuller; the animal regains its appetite and shows all the symptoms of returning health.

In rare cases the disease may be arrested in the stage of red hepatization. In these abortive cases the disease may last only two to three days.

In fatal cases, especially in cases of the typhoid variety, symptoms of general blood dissolution manifest themselves. Œdema of the lungs sets in and death, due to heart failure, comes on.

Prognosis. The prognosis of croupous pneumonia is favorable in a large majority of cases. Unfavorable cases are those presenting the typhoid state and those in which complications develop, especially pleurisy with effusion.

The termination is either in complete resolution, delayed resolution, in chronic interstitial pneumonia (which occurs either in very young or very old animals—those debilitated by some previously existing disease, as bronchitis), by abscess and by gangrene of the lungs. The latter terminations are rare.

Diagnosis. Croupous pneumonia can be readily diagnosticated from catarrhal pneumonia by its clinical course. The sudden onset, the high fever, the physical signs, the termination are all essentially different. The course of catarrhal pneumonia is atypical; you have a history of a preceding bronchitis or a toxic agent inhaled, or food particles getting into the air passages (deglutition or inhalation pneumonia); the fact that catarrhal pneumonia is a double-sided disease, and the spots of dulness on percussion will be found to be small, difficult to make out, and affect only a lobule of the lung. The râles do not disappear in catarrhal pneumonia, but remain throughout the disease; nor is the breathing distinctly bronchial. In catarrhal pneumonia we have subcrepitant râles heard both on in and expiration, whereas in croupous pneumonia we hear a moist râle, which is heard only at the end of inspiration.

The diagnosis from pleurisy can frequently not be made, as I have already indicated that both fibrinous and pleurisy with effusion are concomitant with this disease. In typical cases the history of the disease, the onset, the fever, the cough, and the physical signs will enable one to come to a correct conclusion. In questionable cases the bacteriological examination of the secretions from the nose and mouth will show the presence of the diplococcus of Fraenkel.

There is no specific treatment for pneumonia. Recent experiments by the brothers Klemperer, of Berlin, on the production of immunity and for the cure of pneumonia with subcutaneous and intravenous injections of large quantities of filtered bouillon cultures or the glycerin extract of the germ has produced some remarkable results. Immunity was produced in animals, lasting for six months, which was transmitted to the offspring born within this period. Still more interesting are their observations upon the cure of the experimentally produced disease. They found that the serum and fluids of the body of an animal which had been rendered immune had the property not only of producing immunity when introduced into the circulation of another susceptible animal, but actually of curing the disease after infection had been in progress for some time. In infected animals with a body temperature of from 40° to 41° C. the fever fell to normal in twenty-four hours after the injection of serum of another animal which possessed immunity. They believe that the pneumococcus produces a poisonous albumin (pneumotoxin) which when introduced into the circulation of an animal causes elevation of temperature and the subsequent production in the body of a substance (anti-pneumotoxin) which possesses the power of neutralizing the poisonous albumin which is formed by the bacteria.

In man they hold that during the pneumonia process there is a constant absorption into the circulation of this poisonous albumin produced by the bacteria in the lungs. This continues until eventually the same antidotal substance is produced in the circulation that has been seen to occur experimentally. It is then that the crisis occurs. The bacteria are neither destroyed nor is their power to produce the poisonous albumin lessened, but the third factor, the antitoxic element, now exists and neutralizes the toxic substances as they are produced. They demonstrate that the serum of the blood of patients after the crisis of pneumonia contained the antitoxic substance and was capable, in a fair number of cases, of curing the disease when injected into the infected animals.

While these experiments are still immature, it is nevertheless a decided advance in therapeutics, and seems to approach as near as possible to a specific plan of treatment. Further experiments in this line will be awaited with interest by the entire profession.

Knowing, then, from our present knowledge of therapeutics, what are the main indications for treatment of this disease? First, the hygienic surroundings and food of the patient, should be carefully looked after, the animal should have an unlimited supply of fresh, cold water from the start, a diet consisting principally of bran mashes, scalded oats (grass when in season is preferable if the animal retains an appetite); but if no desire is evinced for food of this particular description, then the animal must be allowed to eat anything that will be taken spontaneously. Corn on the cob is often eaten when everything else is refused. If the horse absolutely refuses to eat, it has been found to be good practice to feed him with oatmeal (preferably Bethlehem oatmeal) and eggs three or four times daily, made into boluses and given in this manner. The comfort and surroundings of the patient must be attended to; pure air is essential. Avoid placing the animal in a stall where he may be exposed to draughts of cold air and sudden changes of temperature. It is considered better practice to blanket the animal than to cut off the fresh air and prevent thorough ventilation.

Locally, mustard, turpentine, etc., have been found useful. In regard to blood-letting, it may be briefly stated that as a systematic course in croupous pneumonia it should not be resorted to. In young, strong animals of good stock, if the case be seen early, and *only* early, local blood-letting has some decided advantages; but in older, more feeble animals, and those affected by some chronic ailments (bronchitis, emphyzematic), it should never be practised. A symptom that often requires special treatment is high fever. This may be treated either locally or constitutionally. Of the local measures, systematic applications of cold water to the chest will be found useful. Another plan is to give large enemata of cold water by the bowel. This will promptly reduce temperature without depressing the heart, and should be used every couple of hours until the temperature is lowered. Experience advises against the use of the analgesic, antipyretics, such as antipyrin, antifebrin, phenacetin, etc., on account of the too depressant effect upon the heart. To add the depressing effect of a powerful drug to the pathological influences already depressing the heart is now recognized as increasing the danger of cardiac failure, which is the most frequent cause of death in croupous pneumonia. While it is true that these drugs unquestionably reduce temperature, they do it at a great risk, and while we have other means of lowering temperature (cold water applications and enteroclysis of cold water), they should not be employed. If any of these drugs be given phenacetin should be chosen, as it has the least depressant effect upon the heart. In reference to the employment of veratrum viride and aconite in the first stage and digitalis at a later period appears as unreasonable. Cardiac depressants in croupous pneumonia are always of doubtful utility, and digitalis as a cardiac stimulant should be given only in response to special indications. Many of the symptoms of pneumonia are due to a toxæmia, and it is far better to bleed the patient, if he is to be bled at all, into a basin than into his own vessels. Later in pneumonia, when the heart becomes weak, digitalis and alcohol are of decided value. Stimulating expectorants during the third stage have some use. The one having the widest reputation and being by far the most used is the carbonate of ammonia. If employed, the dose should be frequently repeated, as the effect of this drug is soon lost. A powerful respiratory stimulant, when such becomes necessary, is strychnia. This should be administered hypodermatically and in full doses. If the cough becomes distressing and painful, resource should be had to opium in some form, preferably as Dover's powder. A favorite plan of treatment in human practice consists in giving full doses of quinine early in the disease, followed by a laxative dose of calomel, reduction of temperature, if it becomes necessary, carbonate of ammonia as a cardiac and respiratory stimulant, control of the fever by means that have already been indicated, digitalis and alcohol if the heart show signs of flagging, and the treatment of complications as they may arise.

The disease being the same, whether it affects man or animal, it seems to me the same treatment might well be practised in veterinary medicine.

It must not be forgotten that many cases of pneumonia will recover without treatment (and also in spite of some treatment), and the less we complicate our case by giving unnecessary drugs the clearer will be the course and the symptoms of the disease.

